# Parsed synthesis of pyocyanin via co-culture enables context-dependent intercellular redox communication

**DOI:** 10.1186/s12934-021-01703-2

**Published:** 2021-11-24

**Authors:** Kayla Chun, Kristina Stephens, Sally Wang, Chen-Yu Tsao, Gregory F. Payne, William E. Bentley

**Affiliations:** 1 Fischell Department of Bioengineering, University of Maryland, 5102 Clark Hall, College Park, MD 20742 USA; 2 Institute for Bioscience and Biotechnology Research, University of Maryland, College Park, USA; 3 Robert E. Fischell Institute for Biomedical Devices, University of Maryland, College Park, USA

**Keywords:** Molecular communication, Co-culture, Pyocyanin, Growth control, Redox, Quorum sensing

## Abstract

**Background:**

Microbial co-cultures and consortia are of interest in cell-based molecular production and even as “smart” therapeutics in that one can take advantage of division of labor and specialization to expand both the range of available functions and mechanisms for control. The development of tools that enable coordination and modulation of consortia will be crucial for future application of multi-population cultures. In particular, these systems would benefit from an expanded toolset that enables orthogonal inter-strain communication.

**Results:**

We created a co-culture for the synthesis of a redox-active phenazine signaling molecule, pyocyanin (PYO), by dividing its synthesis into the generation of its intermediate, phenazine carboxylic acid (PCA) from the first strain, followed by consumption of PCA and generation of PYO in a second strain. Interestingly, both PCA and PYO can be used to actuate gene expression in cells engineered with the *soxRS* oxidative stress regulon, although importantly this signaling activity was found to depend on growth media. That is, like other signaling motifs in bacterial systems, the signaling activity is context dependent. We then used this co-culture’s phenazine signals in a tri-culture to modulate gene expression and production of three model products: quorum sensing molecule autoinducer-1 and two fluorescent marker proteins, eGFP and DsRed. We also showed how these redox-based signals could be intermingled with other quorum-sensing (QS) signals which are more commonly used in synthetic biology, to control complex behaviors. To provide control over product synthesis in the tri-cultures, we also showed how a QS-induced growth control module could guide metabolic flux in one population and at the same time guide overall tri-culture function. Specifically, we showed that phenazine signal recognition, enabled through the oxidative stress response regulon *soxRS,* was dependent on media composition such that signal propagation within our parsed synthetic system could guide different desired outcomes based on the prevailing environment. In doing so, we expanded the range of signaling molecules available for coordination and the modes by which they can be utilized to influence overall function of a multi-population culture.

**Conclusions:**

Our results show that redox-based signaling can be intermingled with other quorum sensing signaling in ways that enable user-defined control of microbial consortia yielding various outcomes defined by culture medium. Further, we demonstrated the utility of our previously designed growth control module in influencing signal propagation and metabolic activity is unimpeded by orthogonal redox-based signaling. By exploring novel multi-modal strategies for guiding communication and consortia outcome, the concepts introduced here may prove to be useful for coordination of multiple populations within complex microbial systems.

**Supplementary Information:**

The online version contains supplementary material available at 10.1186/s12934-021-01703-2.

## Background

Metabolic engineers and synthetic biologists are currently investigating novel strategies to both expand the repertoire of molecular products synthesized in microbial systems and to enhance the production of established products [1]. One strategy that continues to gain traction is the use of microbial co-cultures or consortia, where the tasks required to produce a molecular product are divided amongst multiple populations, rather than being carried out by a single population [2, 3]. Implementation of this strategy allows for division of labor between populations and modularity, where host strains can be optimized for specific tasks [4]. Partitioning a product pathway among different populations also enables culture composition as a control parameter beyond traditional targeting of transcription or translation. To utilize this advantage, development of new methods that allow for robust regulation and coordination of subpopulations for functional control of the whole consortia is necessary.

Previously, we developed a genetic “growth control” module that allowed for autonomous modulation of cell growth rate and culture composition in response to a quorum sensing signal [5]. In our system, upregulation of HPr, a protein involved in sugar transport, in a *ptsH* (encoding HPr) mutant host strain resulted in increased cell growth rate. In a recent example, Dinh et al. developed a co-culture for the production of naringenin, where the growth rate of the population responsible for the earlier part of the pathway autonomously decreased after reaching a certain cell density [6]. Similarly, others have regulated the composition of subpopulations in a microbial community by regulating production of lysis proteins or toxins [7, 8]. These strategies have only just begun to be applied to co-culture systems that are cooperatively synthesizing a molecular product. That is, while there are now many examples of co-cultures being used to synthesize products [9–14], there are few examples with modulated coordination of culture composition and activity.

In our previous work, bacterial quorum sensing signaling has provided the molecular basis for enabling intercellular communication and control. While quorum sensing methodologies can offer cell-specific targeting, particularly through acylated homoserine lactone (AI-1) signaling, this is not always the case. For example, in autoinducer-2 (AI-2) QS systems, signal perception is context dependent; both the specific signal transduction mechanisms and the concentrations that permit collective behavior, can vary based on species [15]. In general, while there is great interest in developing orthogonality in molecular signaling, signal perception and cellular responses are often context dependent. As noted, a cell’s response to the same molecule, even at the same concentration, might result in completely different cell trajectories in different environments. The creation, propagation, and perception of signals for modulating co-cultures and more complex consortia is non-trivial, context dependent, and of great interest.

**Fig. 1:**
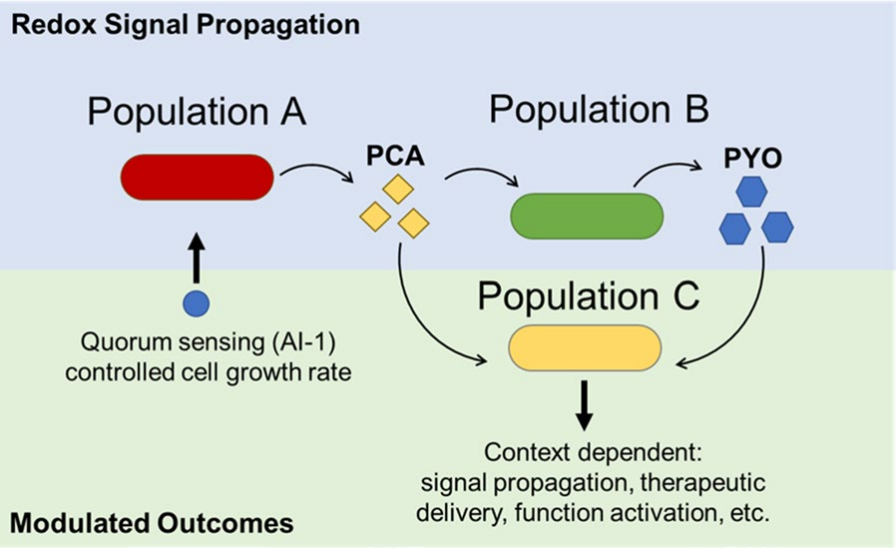
Redox-active phenazine signaling system with quorum sensing regulated growth control module enables context dependent coordination. Parsed synthesis of PYO between Populations A and B enable context dependent activation of Population C in a triculture based on relative composition of the strains. Population A contains a quorum sensing autoinducer—activated growth control module wherein AI-1 (blue dot) activates HPr expression, allowing for tuning of relative populations within the consortia for an additional control parameter for subsequent activation of Population C

Here we demonstrate the signaling capabilities enabled by the redox-active phenazine, pyocyanin (PYO). We developed a robust methodology to produce pyocyanin by separating its synthesis pathway into two strains; one which produces its intermediate phenazine-1 carboxylic acid (PCA) and another which converts this intermediate into PYO (Fig. 1). We characterized this two-strain phenazine producing system’s ability to produce signals, the perception of which are dependent on the prevailing environment. We assessed this co-culture’s ability to propagate signal within a complex microbial community (> two strains), where a third strain’s activity is guided by each of the other strain’s relative molecular contributions. To provide further guidance within multipopulational cultures and to explore coordination between QS and redox based signaling, we overlayed QS-directed compositional control (via AI-1 signal-dependent expression of HPr) by stimulating the growth rate of Population A. When used within the phenazine signaling system, we showed it can guide outcomes of a redox responsive third strain within a tri-culture. We tested this growth control strategy in both rich and minimal media, demonstrating its ability to work with different environments to drive various outcomes.

The relative paucity of orthogonal quorum sensing systems has been cited as a limiting factor in engineering synthetic microbial consortia [16]. Importantly, our design integrates redox-responsive molecules with quorum sensing signals, contributing to diversity of signaling interfaces through which synthetic biologists can exploit for controlling cell populations. We showed that the two strain phenazine producing system could induce production of two marker proteins and a quorum sensing signal synthase in a third strain within a multi-population culture, demonstrating potential for coordinating various activities within a complex culture. We also employed user-applied quorum sensing signal (AI-1) to interface with the phenazine signaling system. Thus, the QS/phenazine system can be used to generate a variety of molecular products and even additional signal molecules, opening new lanes of molecular cell–cell communication. Our signal-actuated growth rate control module was successfully ported into various systems, showing how population control can be effectively embedded into complex signaling contexts. In sum, we demonstrate implementation of parsed PYO biosynthesis for signal propagation in multi-population systems and develop additional methods of coordination by use of a user-directed growth control module to guide redox signaling in various environments [5].

## Results

### *E. coli* strains expressing *phzA1-G1 *(Population A) and *phzMS *(Population B) differentially activate *soxRS* based on environment

To engineer a co-culture that synthesizes pathway intermediate PCA and ultimately pyocyanin (PYO), we split the overall pyocyanin synthesis pathway between two populations. *Pseudomonas aeruginosa* genes *phzA1B1C1D1E1F1G1* were expressed in Population A and genes *phzMS* were expressed in Population B. Mavrodi et al. accomplished this previously in *E. coli* to assist with determining the genetic pathway for pyocyanin synthesis [17]. In that study, the authors showed that the co-culture synthesized pyocyanin (verified by HPLC), but they did not investigate the effect of culture composition on pyocyanin synthesis.

**Fig. 2:**
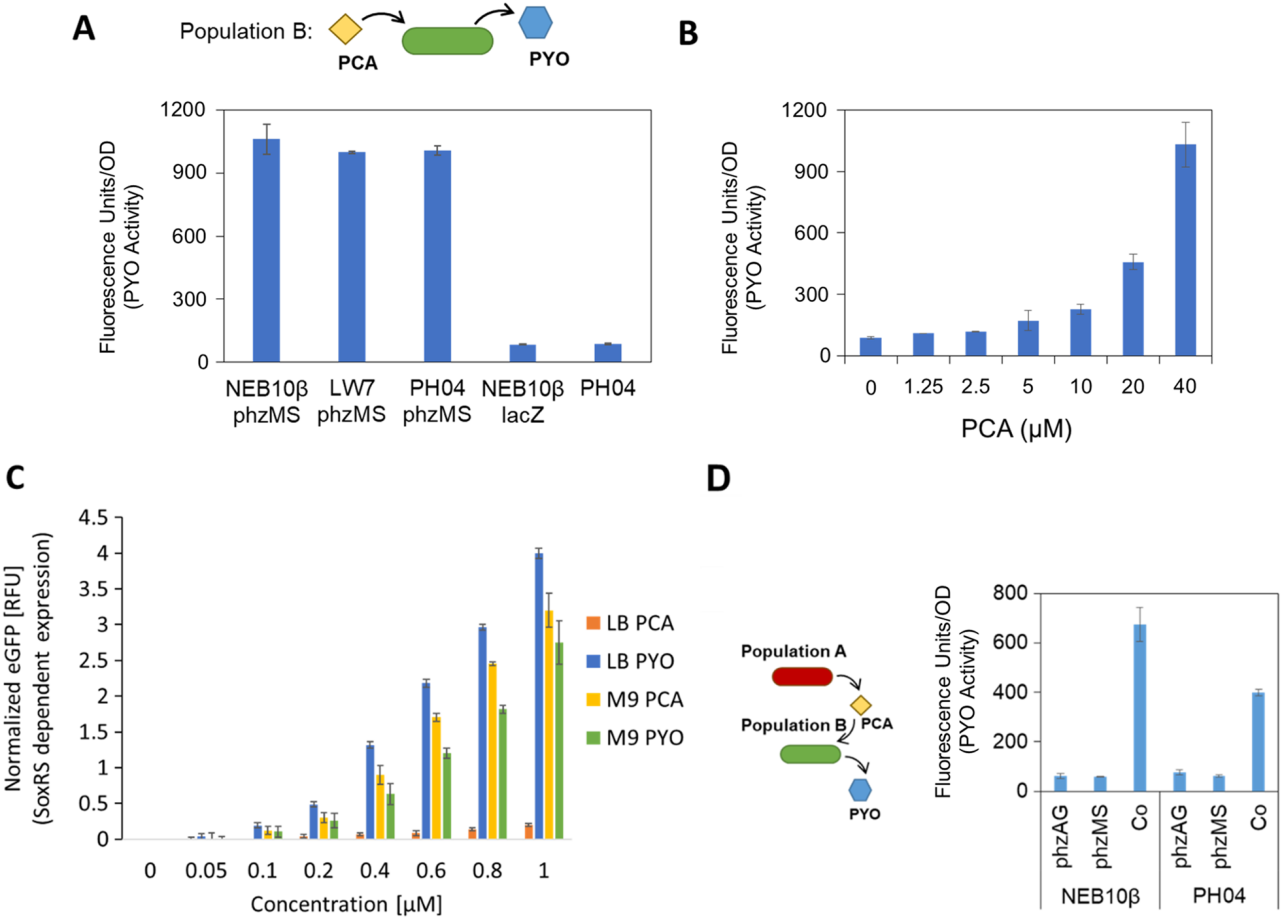
Parsed PYO synthesis enables context dependent activation of *soxRS* induced gene expression. **a** NEB10β, LW7, and PH04 host strains containing pZE-phzMS, pZE-lacZ, or no plasmid (as indicated) were grown in M9 media with 30 µM PCA. Cultures were inoculated in M9 1% (to approximately 0.05 OD_600_) from overnight cultures and PCA was added 1.5 h after inoculation. After overnight growth, CM was collected and tested in a PYO reporter assay using fluorescent reporter cells (SW101 pCT10 pET-DsRedExpress2). The reporter cells were grown to approximately OD_600_ 0.2 in LB media, and 20 µL CM were added to 180 µL reporter cells. Fluorescence was recorded after approximately 4 h. The values reported are the relative fluorescence units divided by the cell density (OD_600_) of the reporter cells. Error bars represent s.d. of technical duplicates. **b** PH04 pZE-phzMS was inoculated 1.5% from overnight cultures. After 1 h, varying amounts of PCA (ranging 0–40 µM) were added. After overnight growth, CM samples were collected. Samples were diluted 5 × in LB media then measured for PYO activity using the reporter bioassay as in **a**. The values reported in **a** and **b** are the RFUs divided by the reporter cells’ OD_600_. Error bars represent s.d. of technical duplicates. **c** PH04 pCT10 pET-eGFP was grown for 4 h during log-phase in either LB or M9 with PYO or PCA at the concentrations indicated (ranging 0–1 µM), then eGFP expression was measured. Fluorescence values were normalized by subtracting out background from reporter cells, then taking its fold over blank. Error bars represent s.d. of technical triplicates. **d** NEB10β pZE-phzAG and NEB10β pZE-phzMS were cultured alone or together (9:1 ratio). Similarly, PH04 pZE-phzAG and PH04 pZE-phzMS were cultured alone or together (9:1 ratio). After overnight growth in M9 media, CM media was collected and assayed for PYO activity in LB media. Error bars represent s.d. of technical duplicates

We first investigated the ability of Population B to convert PCA to PYO. Several host strains were transformed with high copy number plasmid pZE-*phzMS* containing *phzMS* under a LacO-1 promoter. 30 µM PCA was added to cultures grown in M9 media and, after overnight growth, cell-free conditioned media (CM) samples were collected (Fig. 2a). We developed PYO reporter cells, *E. coli* SW101 pCT10 pET-DsRedExpress2 (SW101-DsRed), to detect the presence of PYO in experimental CM samples. These reporter cells were built on our previous work, wherein pyocyanin was used to control expression of a gene of interest under the *soxS* promoter [18]. The genetic construct in that work consisted of *soxR*, the bidirectional *soxRS* promoter, and the gene of interest. Pyocyanin can influence the oxidative state of SoxR, which in turn regulates transcription from the *soxS* promoter. Here, we built a two plasmid system that amplifies expression from the *soxS* promoter, a strategy we used previously to amplify expression from other promoters [19]. A single copy plasmid contains *soxR,* the *soxRS* promoter and T7 RNA polymerase downstream of the *soxS* promoter (pCT10)—a high copy plasmid contains a fluorescent reporter under control of the T7 promoter (pET-DsRedExpress2). In this way, the original promoter strength is amplified by the second promoter activated by the product of the first, T7 RNA polymerase. To detect the presence of PYO in our experimental CM samples, the reporter cells were grown in LB media to approximately OD_600_ 0.2 and CM sample was added. Fluorescence (indicating pyocyanin activity) was measured after approximately 4 h. As indicated, all strains containing *phzMS* converted PCA to PYO (Fig. 2a). Equally importantly, reporter cells in LB media did not respond to CM from strains that did not express *phzMS* but were cultured with PCA. We then tested the ability of strain PH04 pZE-phzMS to convert varying levels of PCA to PYO. We grew PH04 pZE-phzMS in M9 media with 0 to 40 µM PCA, and after overnight growth, collected CM. We added CM to the fluorescent reporter cells in LB media, to determine relative PYO activity (Fig. 2b). Increasing PCA levels resulted in increasing PYO synthesis. At the cell densities and concentrations tested, there did not seem to be a factor limiting PCA conversion, even at the higher concentrations tested.

As noted above, in earlier work we discovered SoxR-responsive elements were actuated by PYO in minimal media [18, 20]. To systematically evaluate signal perception and product formation in response to media context, we carried out experiments with cells expressing SoxR-regulated eGFP in both M9 minimal media and LB media supplemented with both the final signaling product, PYO, and its intermediate, PCA (0–1 mM, Fig. 2c). Additionally, we tested products DsRed and QS signal, AI-1, as the reporters of *soxRS* activity for later use in tri-culture experiments (Additional file 1: Fig. S1). As expected, in Fig. 2c, the level of activation from PYO was high in M9 and was dependent on the supplemented PYO concentration in samples taken after 4 h of incubation. Notably, the level of *soxS* activation from PYO was even higher when cells were cultivated in LB media. Similarly, the level of *soxS* activation was directly proportional to the concentration of PYO added to the LB media (not shown here). However, PCA had almost no stimulatory activity when cells were grown in LB media but led to high levels of *soxS* promoter activity in M9 media; even higher than PYO at the same levels. These results demonstrate that in M9 media, both PCA (the product of Population A) and PYO (the product of Population B) can activate eGFP expression within our sensing Population C. They also demonstrate that in LB media, only PYO (the product of Population B) activates eGFP expression in our sensing Population C. Moreover, in no case was the *soxS* response the same in any media, given the same concentration of stimulant (PYO or PCA). That is, the biological context influenced the responses of Population C to the various signaling molecules.

We then tested whether co-culturing the Population A strain with Population B resulted in PYO production, thus enabling signal propagation. Population A contained plasmid pZE-phzAG comprising *phzABCDEFG* under a LacO-1 promoter. We grew Population A (*E. coli* NEB10β pZE-phzAG) and Population B (*E. coli* NEB10β pZE-phzMS), both alone and together in M9 media (Fig. 2d). Similarly, we grew *E. coli* PH04 pZE-phzAG and *E. coli* PH04 pZE-phzMS, both alone and together in M9 media. Importantly, PH04 is a *ptsH* knockout strain, which facilitated later addition of the growth control module [21]. After overnight growth, we took cell-free CM from the cultures and tested for extracellular PYO using the fluorescent reporter cell assay described earlier (performed in LB media). Each of the co-cultures resulted in high levels of DsRed fluorescence while the monocultures did not, indicating only the co-cultures were able to produce signal in LB media via PYO production.

### Initial co-culture composition of Populations A and B alter signal propagation in tri-culture systems to modulate behavior of a phenazine sensitive strain

**Fig. 3:**
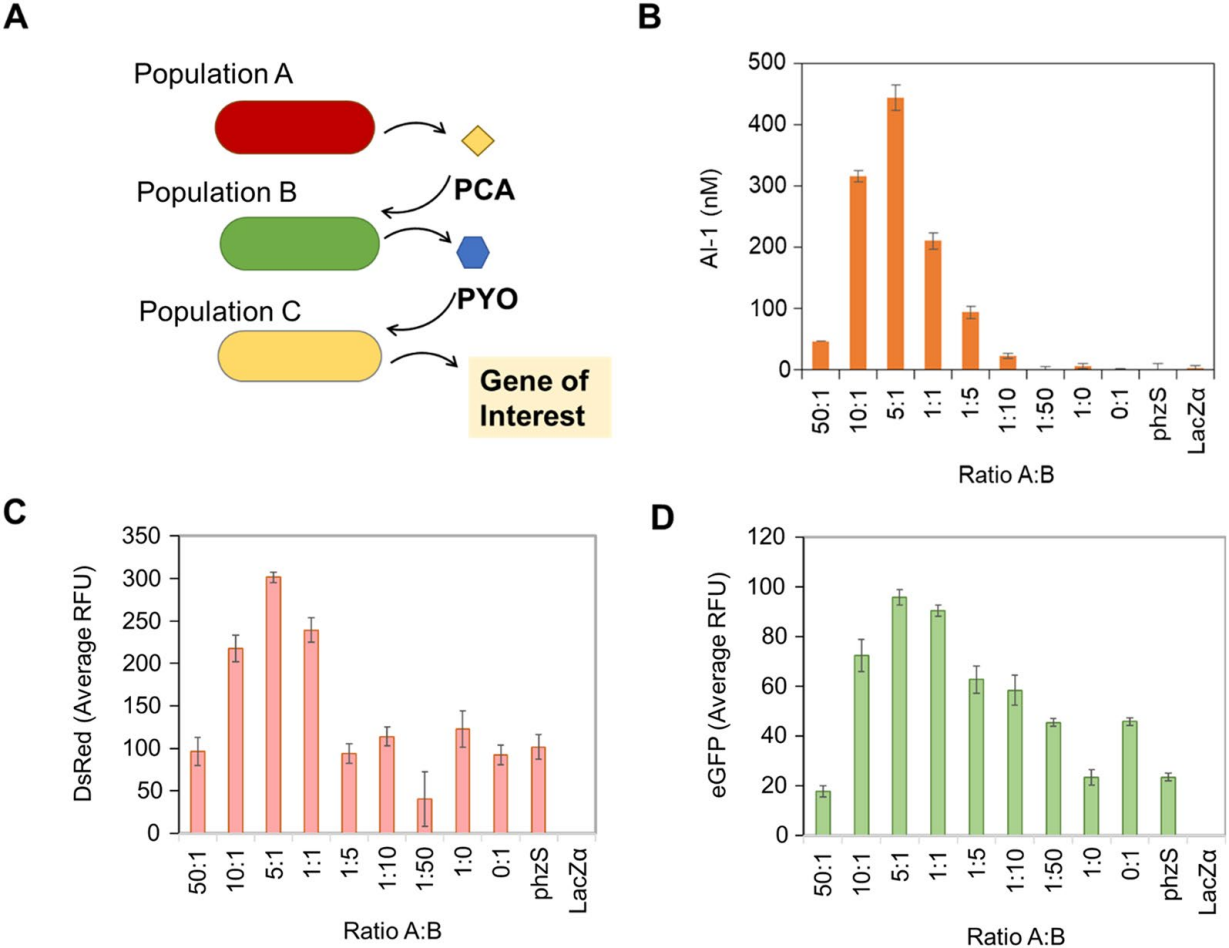
Initial co-culture composition influences activity of a phenazine sensitive strain within tri-culture system in LB. **a** Schematic of tri-culture system in LB media, where PYO drives activity in phenazine sensitive strain, Population C. **b** NEB10β pZE-phzAG (Population A) and NEB10β pZE-phzMS (Population B) were co-cultured in LB media. After 5 h of co-culture, 20 µL of an overnight culture of PH04 pSox-LasI (Population C) grown in LB media were added to the co-cultures. After 1 additional hour of culture, the CM samples were taken. An AI-1 reporter assay was used to measure the extracellular AI-1 levels in the culture.^5^ The controls “phzS” and “LacZα” indicate growth of monocultures NEB10β pZE-phzS or NEB10β pZE-lacZα, respectively, prior to addition of Population C. Error bars indicate s.d. of technical duplicates. **c** and **d** NEB10β pZE-phzAG (Population A) and NEB10β pZE-phzMS (Population B) were co-cultured in LB media. After 5 h of co-culture, 20 µL of the co-culture was added to 180 µL of PH04 pCT10-pET-DsRedExpress2 or PH04 pCT10-pET-eGFP (Population C), **c** and **d** respectively, which were regrown from overnight culture to ~ 0.2 OD_600_ in LB media. After 4 h of additional culture relative fluorescence was measured. The controls “phzS” and “LacZα” indicate monocultures of NEB10β pZE-phzS or NEB10β pZE-lacZα, respectively, in addition to Population C. Background fluorescence was subtracted out from samples by subtracting LacZα value from each sample. Error bars indicate s.d. of technical triplicates

To validate the phenazine system’s signal propagation in a higher order multi-population environment, we tested adding phenazine sensitive strains directly to the two-strain phenazine producing culture (Fig. 3a). We grew *E. coli* NEB10β pZE-phzAG (Population A) and *E. coli* NEB10β pZE-phzMS (Population B) in LB media. We started with different initial ratios of Population A to Population B with total OD_600_ being around 0.1. After 5 h of growth, we added 20 µL of Population C (PH04 pSox-LasI) culture that had grown overnight in LB media. This third population produces AI-1 in response to PYO. Here, AI-1 serves as a model product which could further communicate by serving as an orthogonal signal in a high order quorum sensing consortium. Only 1 h after adding Population C, we took samples and measured AI-1 activity in the extracellular media (using a previously developed AI-1 reporter bioassay) [5, 22]. The results (Fig. 3b) show that Population C responded to PYO in the co-cultures by producing AI-1. As anticipated, cultures missing either Population A or Population B did not result in AI-1 production by Population C in LB media since these populations cannot produce PYO on their own. Importantly, the initial ratio of Population A to Population B was found to greatly influence the production of AI-1 with 5:1 proving to be the optimal ratio.

We additionally tested the system’s ability to influence other types of outcomes in tri-culture by swapping *E. coli* PH04 pSox-LasI Population C for other phenazine sensitive strains. That is, we wanted to verify that the redox-active signal molecules could be used to activate genetic responses for a variety of products. In Fig. 3b, we showed redox-actuated synthesis of LasI and corresponding AI-1 secretion. For additional tests we set up the same co-cultures of Populations A and B as described previously for AI-1 production using different initial ratios. Then we regrew overnight cultures of *E. coli* PH04 pCT10-pET-DsRed and *E. coli* PH04 pCT10-pET-eGFP, which produce protein markers DsRedExpress2 and eGFP in response to *soxS* promoter activation respectively, in fresh LB media to test as Population C. After 5 h of culture, 20 µL of the phenazine producing co-cultures were added to 180 µL of either the *E. coli* PH04 pCT10-pET-DsRed or the *E. coli* PH04 pCT10-pET-eGFP at ~ 0.2 OD_600_. The resultant tri-cultures were then incubated at 30 ℃ for 4 h and then measured for its respective fluorescence (Fig. 3c, d). The results indicated the fluorescence signals were again produced via similar dependencies on initial culture composition as the AI-1 producing tri-culture, where a peak signal was exhibited at the 5:1 ratio. Interestingly, these protein producing cultures required longer induction times for signal propagation to yield its final outcome. This is likely due to longer maturation times for these non-enzymatic fluorescent markers, demonstrating the notable differences Population C can have on processing the phenazine signal into downstream effects dependent on its desired activity.

These results demonstrate the phenazine system’s capabilities of cell–cell signaling within complex synthetic systems (> two strains). That is, in addition to frequently used quorum sensing molecules (ie., AHLs and AI-2), phenazines can elicit specific gene expression via the SoxRS regulon dependent on its environment that can be coopted for additional functions [18, 20]. PYO serves its native host, *P. aeuroginosa*, in several ways, including as a toxin and as a signaling molecule [23]. Here we show that phenazine producing messenger cells and phenazine responding receiver cells can be developed from common laboratory *E. coli* strains for signal propagation and consortium coordination, complementing previous development of *E. coli* strains engineered to produce high levels of PYO [24]. Also, like other ported signaling transduction motifs, should there be a desire to minimize off-target effects stemming from PYO and PCA signaling, hosts can be generated as SoxRS deletion mutants or use multiplexed CRISPR-mediated downregulation [18, 20]. These data also indicate that the tri-culture outcomes varied based on the initial ratio of Population A to Population B, demonstrating that the culture composition affects the rate of biosynthesis and subsequent communication. This allows for utilization of initial A:B ratio as a tunable design parameter for altering activity of the third population.

### Growth control module for user-modulated growth rate in Populations A and B

**Fig. 4:**
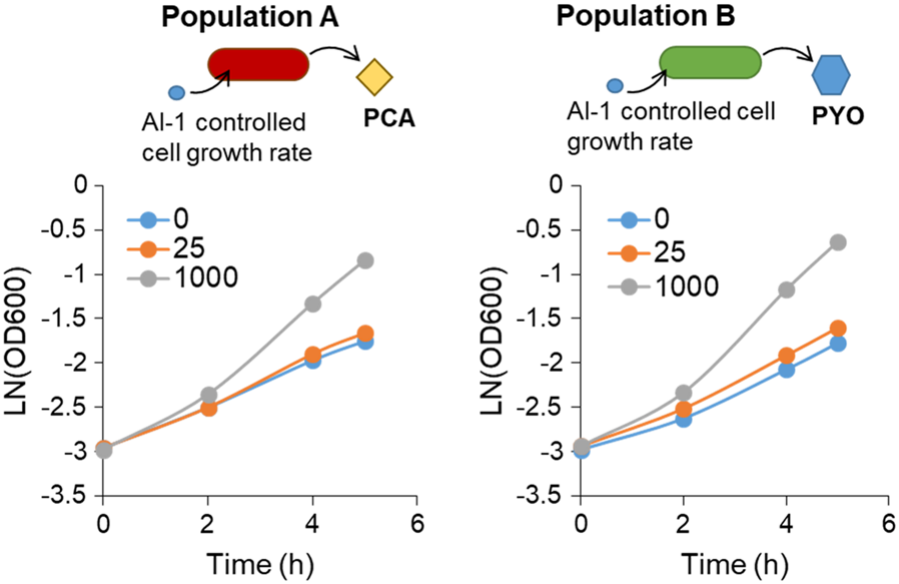
Growth control module enables regulation of cell growth rate in Populations A and B. PH04 pZE-phzAG-ptsH (left panel) and PH04 pZE-phzMS-ptsH (right panel) were grown in M9 media with glucose. Either 0, 25, or 1000 nM AI-1 (as indicated) were added at *t* = 0. Cell density was recorded over time, the plots represent one replicate

Having demonstrated that *E. coli* co-cultures expressing *phzAG* and *phzMS* produce PYO in both M9 media and LB media, and that the culture composition affects phenazine synthesis and signal propagation, we next sought to add our previously developed growth control module to either Population A or B in order to allow for user-modulated cell growth rate of either subpopulation [5]. The growth control module contains *ptsH* under the *lasI* promoter and *lasR* under a constitutive T5 promoter. As a population marker, DsRedExpress2 is also constitutively expressed. In this system, AI-1 addition upregulates HPr (encoded by *ptsH*), which increases cell growth rate in a *ptsH* mutant host strain [5, 21]. Here, we added these components to the pZE-phzAG and pZE-phzMS plasmids to create plasmids pZE-phzAG-ptsH and pZE-phzMS-ptsH. We transformed the *ptsH* mutant strain *E. coli* PH04 (see Figs. 2 and 3) with these plasmids. We then grew *E. coli* PH04 pZE-phzAG-ptsH and *E. coli* PH04 pZE-phzMS-ptsH (separately) in M9 media and tested whether AI-1 addition changed the culture growth rate (Fig. 4). As anticipated, increased AI-1 levels resulted in increasing cell growth rate. Notably, the calculated growth rates between 2 and 5 h after AI-1 addition increased from 0.25 h^−1^ and 0.28 h^−1^ for *E. coli* PH04 pZE-phzAG-ptsH and PH04 pZE-phzMS-ptsH, respectively, with no AI-1 to 0.51 h^−1^ and 0.57 h^−1^ for *E. coli* PH04 pZE-phzAG-ptsH and PH04 pZE-phzMS-ptsH, respectively, with 1000 nM AI-1 effectively doubling the growth rate of these cells. The difference in growth rate between cultures grown with or without AI-1 is similar to what was previously observed [5] (in cells without the *phz* operon genes), and therefore the expression of the *phz* operon genes did not appear to interfere with the function of the growth control module in either strain tested. These results further demonstrate the portability of the previously described HPr growth controller to cells that have different functions, each synthesizing a part of a metabolic biosynthesis pathway towards pyocyanin.

### Tuning growth rate in Population A modulates outcomes of the phenazine signaling system

**Fig. 5:**
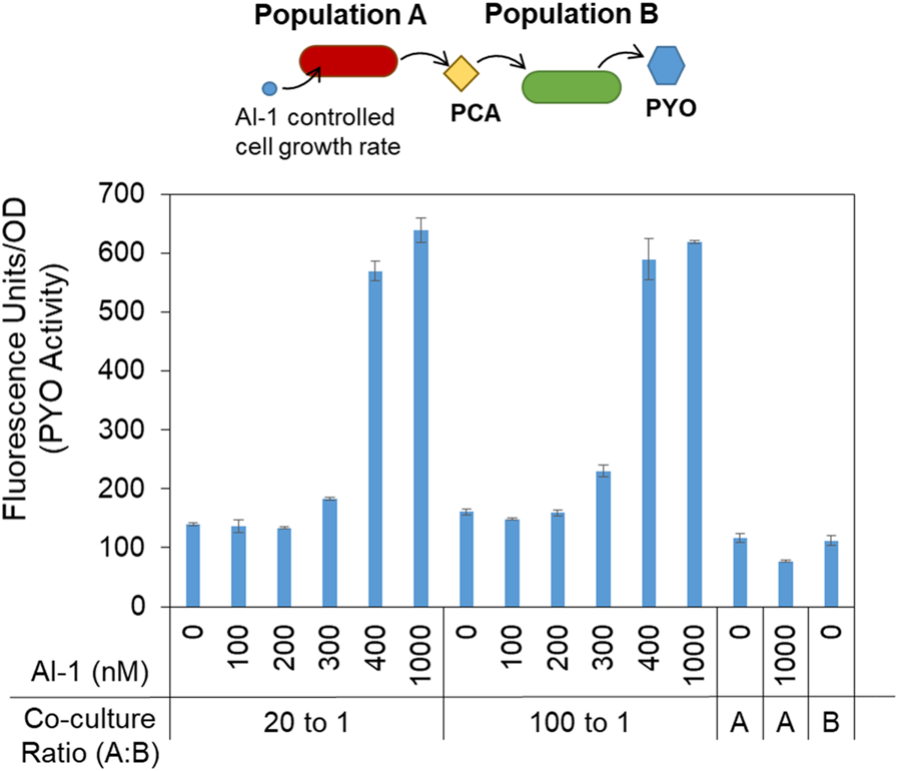
Signal output from growth control module toggles phenazine signaling co-cultures in LB media. PH04 pZE-phzAG-ptsH and PH04 pZE-phzMS were co-cultured together in M9 media with the initial inoculation ratios, as indicated. Population A contains pZE-phzAG-ptsH and exhibits AI-1 modulated growth rate. One hour after inoculation, the indicated concentration of AI-1 was added. The cultures were grown overnight. Subsequently, CM samples were collected and PYO activity was tested using the fluorescent reporter assay in LB media (see Methods). Error bars represent s.d. of technical duplicates

We then tested co-cultures of Populations A and B, wherein we changed Population A so that it contained the HPr growth control module. We grew Population A (*E. coli* PH04 pZE-phzAG-ptsH) and Population B (*E. coli* PH04 pZE-phzMS) in M9 media using different initial ratios of A to B (20:1 and 100:1). As before, we ran these experiments with a total starting OD_600_ of ~ 0.1, and then added different concentrations of AI-1 1 h after inoculation. After overnight growth, we collected conditioned media to test for extracellular pyocyanin using the fluorescent reporter assay in LB media such that any residual PCA is not recorded. Figure 5 shows the fluorescence, indicative of PYO, as measured by the reporter cells (*E. coli* SW101-DsRed). Increased AI-1 levels increased the level of PYO produced in the overnight co-cultures. The fold change in PYO produced was nearly fivefold between the cultures without AI-1 and with 1 µM AI-1. Interestingly, this fold change was found irrespective of the initial culture ratio of A to B, suggesting that the resultant PYO synthesis was influenced most heavily by the AI-1 addition (which modulates Population A growth rate). Here, AI-1 serves as additional knob to alter phenazine signaling via PYO production. Without the growth control module (Fig. 2d) both populations are required to produce PYO, whereas here both populations plus AI-1 seem to be required for significant signal production. We suspect that the growth control module, which includes constitutive expression of DsRedExpress2 and LasR, may divert some resources away from PCA synthesis, and this effect of constitutive expression of DsRedExpress2 and LasR on PCA synthesis may be especially noticeable when *ptsH* is not induced (when there is no AI-1). This could explain why the co-cultures with the inducible growth control module in Population A produce little PYO when there is no AI-1, while the co-cultures without the growth control module (which do not have constitutive expression of DsRed) do produce PYO. The basal level of *ptsH* and/or the metabolic activity of Population A might be important. Consistent with this notion, by comparing Fig. 5 with Fig. 2d, when AI-1 is used to induce *ptsH* expression (i.e., increase Population A metabolic activity), we found an increase in fluorescence indicating increased PYO compared to co-culture without the growth control module.

### Growth control module enables user control of signal propagation in tri-cultures

After demonstrating the growth control module’s effect in our phenazine signaling co-culture, we tested capabilities of user control to modulate activity in a multi-population system. We grew Population A with the HPr growth controller and DsRed expression (*E. coli* PH04 pZE-phzAG-ptsH-DsRed) and Population B (*E. coli* PH04 pZE-phzMS) that converts PCA to PYO in either M9 or LB media at various initial composition ratios and AI-1 concentrations. Then, after overnight growth, we re-grew the co-cultures in fresh media and supplemented with Population C, the same phenazine sensitive strain as used in Fig. 3d (*E. coli* PH04 pCT10-pET-eGFP) that produces eGFP, to easily monitor activity via fluorescence. In all cases, we added Population C at roughly the same OD as the total OD of Populations A and B. We tested these tri-cultures in both M9 and LB mediums to assess the effects of environment on the growth control module’s capabilities. In these experiments, we expected to see eGFP expression in Population C to increase with initial AI-1 concentration and initial Population A composition. In both cases, we hypothesized increased Population A would lead to increased PCA and PYO, leading to stronger signal propagation and higher eGFP expression. We also expected to see little or no fluorescence in Population C from cultures without Population A (i.e. Population A is required to produce intermediate, PCA, and downstream product, PYO).

**Fig. 6:**
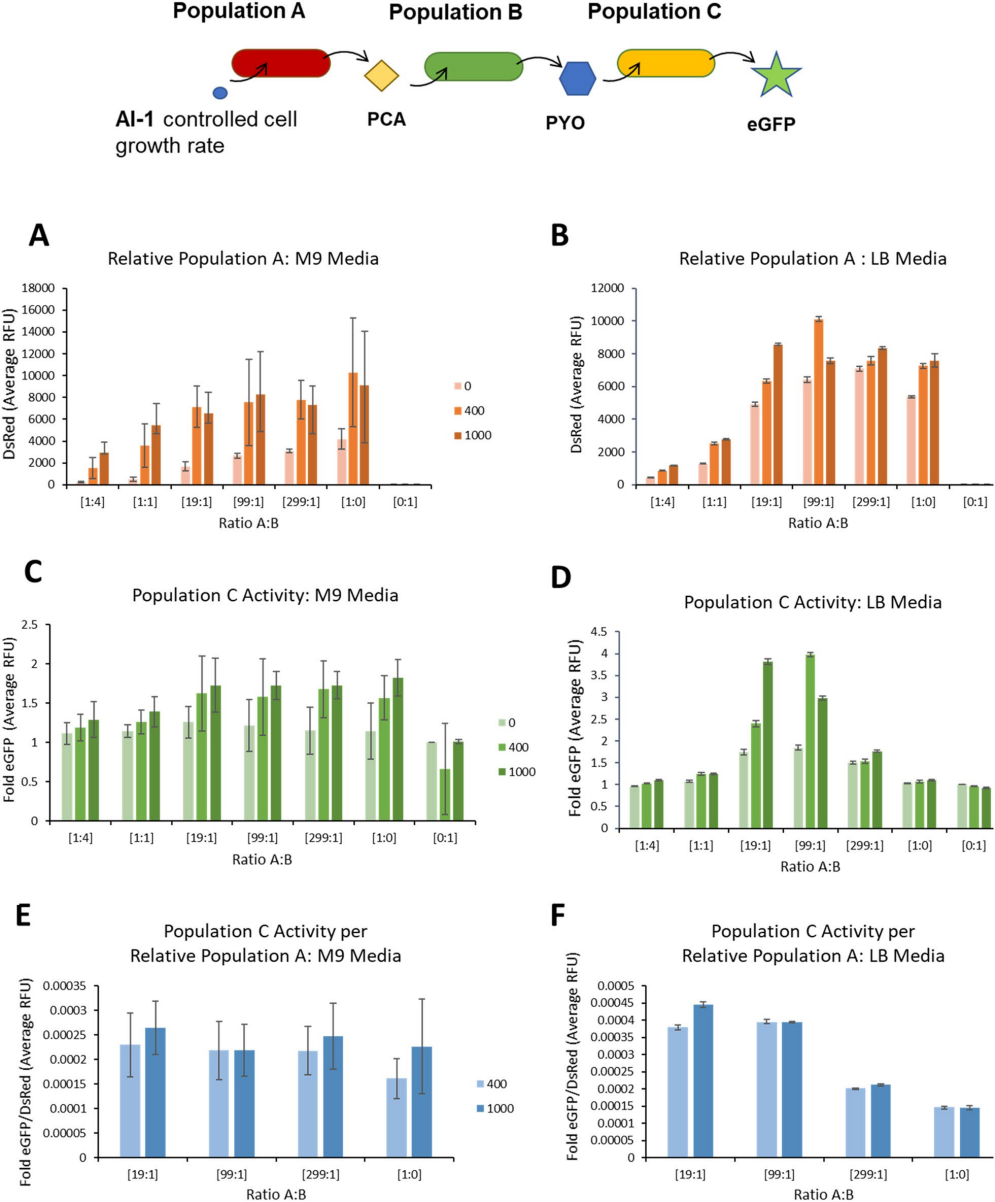
Population A growth modulates Population C activity dependent on initial co-culture composition, AI-1 addition, and environment. Population A (*E. coli* PH04 pZE-phzAG-ptsH-DsRed) and Population B (*E. coli* PH04 pZE-phzMS) were co-cultured together at initial inoculation ratios as indicated on the x-axis and with nanomolar AI-1 concentrations as indicated in the legend. After overnight growth, 20 µL of the PYO producing co-culture (~ 1.5 OD_600_) was added to a culture of ~ 0.2 OD_600_ Population C (PH04 pCT10-pET-eGFP) with 200 µL final volume in fresh media. The tri-strain culture was then incubated for 4 h, and then measured for DsRed and eGFP fluorescence. **A**, **B** Raw DsRed fluorescence of the tri-strain co-culture at 4 h, indicating Population A composition in culture. **C**, **D** Average eGFP fluorescence fold over background of tri-strain co-culture, indicating Population C activity. **E**, **F** Average eGFP divided by average DsRed, indicating relative Population C activity per Population A. Error bars indicate s.d. of triplicates

After 4 h of tri-culture growth, we measured DsRed and eGFP fluorescence of Population C to assess the co-culture’s effect on the total system. Figure 6a, b shows average raw DsRed fluorescence, indicative of relative amounts of Population A in the multi-population culture. Figure 6c, d shows average eGFP fluorescence fold over background that is attributed to Population C activity. In both M9 and LB media, the initial ratio appeared to influence the composition and eGFP measured in cultures without AI-1, particularly when there were more Population A cells to begin with. These results are consistent with Fig. 3, as we found there that initial composition affects signal propagation in tri-culture. In Fig. 6a–d, we also demonstrated that stimulation of Population C (eGFP expression) in a tri-strain culture is correlated to the relative quantity of Population A in culture as indicated by total DsRed fluorescence. Thus, we found here that one can alter the consortium by increasing AI-1 concentration to turn on the growth control module, which increases both the relative amounts of Population A and eGFP expression in Population C, regardless of the initial ratio of Population A to B.

It was interesting to note that in both M9 and LB media that as the ratio of A:B was increased, the relative fluorescence of Population C increased, reaching an apparent maximum at the highest AI-1 levels when the fraction was between 19:1 and 99:1, suggesting that the influence of the altered growth rate was at a maximum in this region (Fig. 6c, d). In both environments, at higher initial ratios without AI-1 there appeared to be a saturation in Population C activity from the additional number of Population A cells.

We also noted that in M9 media, at initial ratios above 19:1, growth rate modulation seemed to have less of an impact in determining composition. Interestingly, while 400 nM AI-1 addition dramatically increased the relative amounts of Population A (DsRed fluorescence), further AI-1 (i.e., to 1000 nM) did not. That said, the additional AI-1 did appear to increase Population C’s eGFP production. We plotted the ratio of eGFP to DsRed to qualitatively assess Population C activity per Population A for high initial ratios and with AI-1 addition (Fig. 6e); our data suggest that Population C activity was typically equal to or a bit higher at 1000 nM AI-1 than 400 nM. Perhaps in M9 media where both PCA and PYO are generated and also influence Population C, the added growth control module activity contributed to signal propagation as well as to the composition of Population A. The 1:0 culture, where additional AI-1 resulted in additional Population C activity (Fig. 6e) reinforces this in that this culture only had Populations A and C and no PYO from B. Thus, the added Population C activity was likely responding to additional PCA made per cell of Population A for producing a signal.

Comparatively, in LB media at ratios above 19:1 and with AI-1 addition, the effects of increased Population C activity actually diminished with increased Population A (Fig. 6f). Here, incremental increases in PCA produced per cell of Population A would not be reflected in Population C activity, which is consistent with the ratios depicted in Fig. 6f. Instead, at high initial ratios and with additional AI-1 where Population A increases without increase in Population C activity, suggests that Population B might be limiting in that there is less conversion of PCA to PYO. Interestingly, we evaluated the growth rates of all tricultures spanning all initial ratios and AI-1 levels (see Additional file 1: Table S3). Naturally, the growth rates in LB media were higher than in M9 media, but in nearly all cases, they were not distinguishable from each other. That is, even while the fraction of Population A was steadily increasing in the presence of AI-1 and when at 19:1 initial ratio to B, the overall growth of the tri-culture was unaffected. We note, however, the control cultures with just Population A and C [i.e. A:B = 1:0], behaved as anticipated. Perhaps our finding that the apparent growth rate of the tricultures was largely unchanged is often the case for complex consortia in complex media. Further studies are currently underway to investigate the dynamic nature of the specific signal molecule levels, their uptake and subsequent temporal effects on gene expression.

## Discussion

In this work, we have demonstrated the potential for combining quorum sensing and redox-based signaling in synthetic consortia. We employed a user-actuated genetic switch to control signaling mediated by pyocyanin synthesis in both two-member and three-member systems. Initially, using a two-member consortium, we employed culture composition as a parameter for controlling metabolic flux and signaling, as opposed to directly regulating transcription in either strain. In a three-member consortium, we used our previously reported growth control module to apply user-controlled quorum sensing communications for guiding cooperative signaling by phenazines PCA and PYO. Interestingly, we also found that the importance of the growth control module was most impactful at initial composition ratios below 99:1 and further found that outcomes were impacted less when initial ratios were high (~ 99:1 to ~ 299:1). That is, the outcome was already strongly determined by the predominance of Population A and further increases were less impactful. We suggest, however, that a strategy of altering the composition and metabolic activity may be broadly applicable in other systems as pyocyanin is derived from the shikimate pathway, a common starting point for many molecular products [25].

Strategies for modulating culture composition and activity may be of even more general utility as metabolic engineers and synthetic biologists consider ways to modulate microbiomes. Importantly for these contextual applications, we found that media composition influenced the *soxR*-driven gene expression, based on whether PCA or PYO was the predominant signaling molecule. For instance, the effect of the relative levels of Population A to B on the behavior of Population C varied based on media. Due to PCA and PYO’s variable influence in *soxRS* gene expression in various hosts (that was shown to be dependent on the growth media), we suggest that context-dependent signaling can be an advantage or a disadvantage but is certainly a factor that needs to be considered for future applications of microbial consortia. Previous studies have also shown consortia outcomes to be dependent on nutrient availability and environmental factors [26, 27]. That is, while it is attractive to consider unique orthogonality among signaling molecules, cultivation conditions and deployment context of consortia must be considered. Temperature, carbon sources, and the presence of other metabolites that may interfere with signal propagation and a consortium’s growth and functional success are important for robust design and optimization during bottom-up assembly. We note, however, that while the cell growth behavior of the tricultures was indistinguishable in this study, the underlying signaling methodologies were retained and were distinguished.

The novel communication strategies demonstrated by this work also implies that user directed PYO producing co-cultures might have advantages for coordinating activity within cultures of higher order populations. We showed conversion between quorum sensing and redox communications by interplaying AI-1 and the PYO synthesizing coculture to influence one another’s signaling. Thus, users might introduce and control a PYO producing co-culture to modulate orthogonal signaling molecules such as quorum sensing AHLs that, in turn, induce activity in downstream strains within a higher order multi-population system (i.e. > three strains). Additionally, the growth control module shown here might enable a finer level of control where initial consortia composition is limited, in that it may influence metabolic flux as well as population composition.

## Conclusions

We showed that the pathway for synthesis of pyocyanin could be split between two populations and guided by initial composition of the consortia and subsequent growth modulation of the PCA producing strain. We further showed that PYO and even PCA could be used in *E. coli* as a molecule for cell–cell communication. By splitting the pathway for PYO synthesis between two populations, the behavior of the phenazine sensitive receiver is dependent on the relative composition of the PYO producing strains. We then demonstrated capabilities for user-directed control of this signal propagation in multi-population cultures by modulating the growth rate and therefore co-culture composition and metabolic activity.

The tools we have developed here support user-operated consortia control, however multi-population systems especially those built from the bottom up, are sensitive to the prevailing balance of population, molecular signaling, metabolic activity, etc., in which environment-specific context must be considered. Irrespective of the particular environment, we were able to use PCA and PYO as signaling molecules to drive downstream activity. We also effectively employed a user-directed quorum sensing induced growth control module to influence intercellular redox communication that propagated signal to guide a third strain’s activity. Moving forward, these tools could be used to modulate a keystone organism of particular importance within a consortium or coordinate higher order activity. Such strategies that facilitate engineered cooperativity in synthetic biology systems may find utility in future applications including those where co-cultures and mini consortia are used for small molecule synthesis.

## Methods

### Strains and plasmids

The strains, plasmids, and primers used in this study are listed in Additional file 1: Tables S1 and S2. Plasmids pZE-phzAG, pZE-phzMS, and pZE-lacZα are derived from the commercial vector pZE12MCS (ExpresSys), a high copy colE1 origin plasmid. Each plasmid contains the genes of interest under the LacO-1 promoter in the vector. The *phzA1-G1*, *phzM*, and *phzS* genes were amplified from *P. aeuruginosa* PA01 using primers KpnI-phzA1-FWD and HindIII-phzG1-RS, HindIII-phzM-FWD and HindIII-phzM-RVS, and KpnI-HindIII-RBS-phzS-FWD and BamHI-phzS-RVS, respectively. The *phzA1-G1* fragment and the pZE12MCS vector were digested with KpnI and HindIII, and ligated to form pZE-phzAG. Plasmid pZE-phzMS was cloned in a multi-step process. When amplifying *phzS* a ribosomal binding site was placed upstream of *phzS* along with KpnI and HindIII restriction digestion sites. A BamHI restriction digestion site was added downstream of *phzS*. This fragment and the pZE12MCS vector were digested with KpnI and BamHI restriction enzymes, and ligated to form pZE-phzS. Then, the *phzM* fragment and pZE-phzS were digested with the HindIII restriction enzyme, and ligated to form plasmid pZE-phzMS.

To construct plasmid pZE-lacZα, *lacZα* was amplified from a wild type *E. coli* strain (W3110 derivative), using primers KpnI-lacZalpha-FWD and BamHI-lacZalpha-RVS. The fragment and pZE12MCS vector were digested with KpnI and BamHI restriction enzymes, and ligated.

Plasmids pZE-phzAG-ptsH and pZE-phzMS-ptsH were generated by adding the growth control module to the pZE-phzAG and pZE-phzMS plasmids. The growth control module, consisting of *ptsH* under the *lasI* promoter and dsRedExpress2 and *lasR* under a constitutive T5 promoter, was amplified from pAHL-HPr [5]. Primers PciI-t7-term-rvs and PciI-ptsH-rvs were used to amplify the control module and add a PciI restriction digestion site on either end of the fragment. Restriction digestion and ligation were used to insert the fragment into the pZE12MCS vector at the PciI restriction digestion site.

Plasmid pCT10 is a low copy plasmid derived from pFZY1 [28] *soxR*, along with the divergent *soxR and soxS* promoters were amplified from *E. coli* using primers Fsoxp and Rsoxp. This fragment was inserted into the Invitrogen pCR-Blunt II-TOPO vector generating plasmid pTOPO-SoxRS. Restriction digestion of plasmids pFZY1 and pTOPO-SoxRS with restriction enzymes BamHI and HindIII, and ligation were used to insert *soxR* and *sox* promoters into pFZYI. T7 RNA polymerase was then inserted downstream of the *soxS* promoter at the HindIII restriction digestion site to generate plasmid pCT10.

Strain SW101 is derived from *E. coli* ZK126 (W3110 Δ*lacU169 tna-2*). The Datsenko and Warner method [29] was used to delete *soxR,* the divergent *soxRS* promoter region, and *soxS*. Primers soxHP1 and soxHP2 were used for the deletion.

### Cell culture conditions

LB media was used for cloning and overnight growth of cultures inoculated from glycerol stocks. M9 minimal media (1 × M9 salts, 2 mM MgSO_4_, 0.1 mM CaCl_2_, 0.2% casamino acids, 0.4% glucose) or LB media were used for experiments as indicated in the figure captions. Cultures were grown at 37 °C with 250 rpm shaking. Ampicillin (50 µg/mL) and/or kanamycin (50 µg/mL) was used to maintain plasmids.

### PYO fluorescent reporter assay

A fluorescent reporter assay was used to quantify PYO in experimental samples. CM samples were collected by filtration through a 0.2 µM filter and stored at − 20 °C until analysis. The fluorescent reporter cells, SW101 pCT10 pET-DsRed, consist of a dual plasmid system. A single copy plasmid contains the pyocyanin sensitive *soxS* promoter. T7 polymerase is under the *soxS* promoter and activates DsRed on the high copy pET plasmid.

For the assay, SW101 pCT10 pET-DsRed cells were reinoculated in LB media from overnight cultures and grown to approximately 0.2 OD_600_. 180 µL of reporter cells and 20 µL of sample were added together in 96 well, black wall, clear bottom plates. Cultures were grown at 30 °C, 250 rpm shaking for approximately 4 h. A SpectraMax M2e plate reader was used to read DsRed fluorescence and OD_600_. For fluorescence, the excitation wavelength was 550 nm, the emission wavelength was 579 nm, and a cutoff of 570 nm was used. The reported value was divided by the cell density (OD_600_).

### AI-1 luminescent reporter assay

To measure extracellular AI-1, cell-free conditioned media samples were collected. *E*. *coli* AI-1 reporter cells with plasmid pAL105 [22] were grown overnight in LB media, and then diluted 2500 fold in fresh LB media. 10 µL of samples were added to 90 µL of reporter cells. If necessary, samples were diluted prior to the assay to be in the linear range of the assay. Samples making up a standard curve of known AI-1 concentrations 0, 12, 24, 36, 48, and 60 nM AI-1 were also added to the reporter cells. After 3 h, luminescence was recorded. A linear fit was used to determine the standard curve, which was then used to calculate the AI-1 in the experimental samples. If the experimental samples were diluted prior to the assay, the results were multiplied by the dilution factor (usually 5 or 10) to back calculate to the AI-1 in the original sample.

### Tri-strain co-culture assay

Population A (PH04 pZE-phzAG-ptsH) and Population B (PH04 pZE-phzMS) were co-cultured together overnight in M9 media with AI-1 concentrations ranging 0–1000 nM. Overnight cultures were incubated at 37 °C at 250 rpm shaking. After overnight growth, 20 µL of co-culture was added to 180 µL of ~ 0.2 OD_600_ Population C (PH04 pCT10 pET-eGFP) culture in 96 well, black wall, clear bottom plates. The tri-strain culture was then incubated for 4 h at 30 °C with shaking in a TECAN Spark plate reader. DsRed (Ex/Em: 550/579 nm), eGFP (Ex/Em: 488/507 nm), and OD_600_ were measured every 30 min to quantify Population A relative composition and Population C activity.

## Supplementary Information

**Additional file 1. **Tables S1, S2, and S3. Fig. S1 and S2.

## Data Availability

All data generated or analyzed during this study are included in this published article and its additional files.

## Abbreviations

AI-1: Autoinducer-1
CM: Conditioned media
PCA: Phenazine-1-carboxylic acid
PYO: Pyocyanin
RFU: Relative fluorescence units
