# Supplementary material for: Parsed synthesis of pyocyanin via co-culture enables context-dependent intercellular redox communication
Source: Microb Cell Fact. 2021 Nov 24;20:215. doi: 10.1186/s12934-021-01703-2 (PMC8611841; doi:10.1186/s12934-021-01703-2)
Supplement: Supplementary file 1 — Additional file 1. Tables S1, S2, and S3. Fig. S1 and S2. [file 12934_2021_1703_MOESM1_ESM.docx]

**Additional file**

**Table S1: Strains and plasmids used in this study**

| **Strains** | **Genotype** | **Source** |
| --- | --- | --- |
| *E. coli* |  |  |
| NEB10β | Δ(ara-leu) 7697 araD139  fhuA ΔlacX74 galK16 galE15e14-  ϕ80dlacZΔM15  recA1 relA1 endA1 nupG  rpsL (Str^R^) rph spoT1 Δ(mrr-hsdRMS-mcrBC) | New England Biolabs |
| PH04 | W3110 ∆*lacU169 tna-2* ∆*luxS* ∆*ptsH* | ^1^ |
| ZK126 | *E. coli* K-12 substr. W3110 Δ*lacU169* *tna-2* | Laboratory Stock |
| LW7 | W3110 ∆*lacU169 tna-2* ∆*luxS*::Kan | ^2^ |
| SW101 | ZK126 Δ*soxRS* | This study |
| **Plasmids** | **Relevant Property** | **Source** |
| pZE12MCS | colEI origin, LlacO-1 promoter followed by multi-cloning site, Ap^r^ | ExpresSys |
| pZE-phzAG | pZE12MCS derivative, containing *phzA1-G1* under LlacO-1 promoter, Ap^r^ | This study |
| pZE-phzMS | pZE12MCS derivative, containing *phzM and phzS* under LlacO-1 promoter, Ap^r^ | This study |
| pZE-LacZα | pZE12MCS derivative, containing *lacZα* under LlacO-1 promoter, Ap^r^ | This study |
| pZE-phzAG-ptsH | pZE-phzAG derivative, containing growth control module (*ptsH* under *lasI* promoter, and dsRedExpress2 and LasR under constitutive T5 promoter) | This study |
| pZE-phzMS-ptsH | pZE-phzAG derivative, containing growth control module (*ptsH* under *lasI* promoter, and dsRedExpress2 and LasR under constitutive T5 promoter) | This study |
| pET-DsRed | pET200 derivative, containing *dsRed_Express_DR* | ^3^ |
| pCT10 | pFZY1, *soxR*, *soxR* and *soxS* intergenic region, T7 RNA polymerase | This study |
| pTT01 | pBR322, *soxR* gene and the overlapping divergent *soxR* and *soxS* promoters, phiLOV downstream of *soxS* promoter, Ap^r^ | ^4^ |
| pSox-LasI | pTT01 derivate, containing *lasI* under *soxS* promoter, Ap^r^ | ^1^ |
|  |  |  |

**Table S2: Primers used in this study**

| **Primer** | **Sequence** |
| --- | --- |
| PciI-t7-term-RVS | TCGTACATGTCAAAAAACCCCTCAAGACCCGT |
| PciI-ptsH-RVS | ATCTACATGTTACTCGAGTTCCGCCATCAG |
| HindIII-phzM-RVS | cctagtAAGCTTTCATCAGGCCCTGGCA |
| HindIII-phzM-FWD | cactgAAGCTTATGAATAATTCGAATCTTGCTGCTGC |
| KpnI-phzA-FWD | tgcatGGTACCATGAACGGTCAGCGGTAC |
| HindIII-phzG1-RVS | CATACAAGCTTCACGGTTGCAGGTAGC |
| KpnI-HindIII-RBS-phzS-FWD | tacgtGGTACCAAGCTTAAAGAGGAGAAAGCACCCATGAGCGAA |
| BamHI-phzS-RVS | aagtaGGATCCTAGCGTGGCCGTTC |
| Fsoxp | GTTCTAGGATCCTTAGTTTTGTTC ATCTTCCAGCAAGCG |
| Rsoxp | GACTTTAAGCTTAAATCTGCCTCT TTTCAGTGTTCAGTTC |
| soxHP1 | GTTCATCTTCCAGCAAGCGTGCGCCGGTACCTTCTTCTCCTAAGCGGTCGGTGTAGGCTGGAGCTGCTTC |
| soxHP2 | CAATGGATGGAGCAATTACCCGCGCGGGAGTTAACGCGCGGGCAATAAAACATATGAATATCCTCCTTAG |

**Figure S1: Product Calibration and Standardization**

**
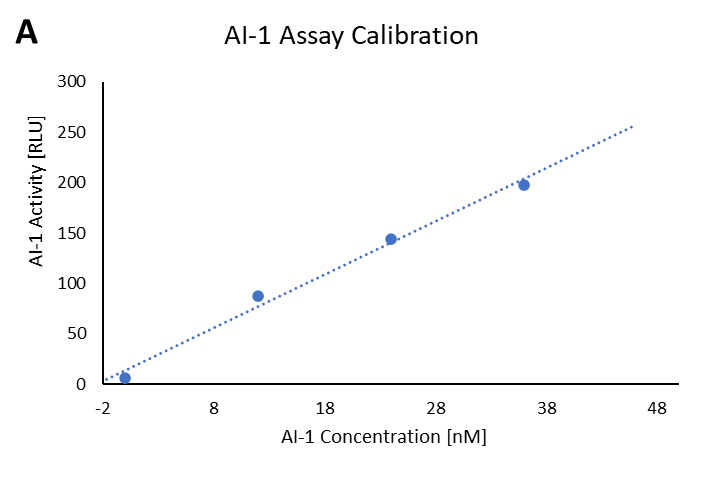
**Population C end products (AI-1, DsRed, and eGFP) were calibrated to standard measurements, then standardized in response to PYO and PCA in M9 and LB media. **a)** Linear region of standard curve from AI-1 luminescent reporter assay as described in Methods. **b-e)** Cultures of PH04 pCT10 pET-eGFP and PH04 pCT10-DsRed were inoculated from an overnight seed culture at a 1:20 ratio in either M9 or LB media, with a concentration of 1µM PYO or PCA. These samples were incubated at 30⁰C overnight, then measured for OD_600_ and fluorescence. **f-h)** PH04 pSox-LasI, PH04 pCT10 pET-eGFP, and PH04 pCT10 pET-DsRed were grown for 4 hours during log-phase in either LB or M9 with PYO or PCA at the concentrations indicated (ranging 0-1 µM), then eGFP, DsRed expression were measured using fluorescence and AI-1 was measured a cell based reporter assay (see Methods). eGFP measurements were normalized by subtracting background fluorescence from uninduced cells, then taking its fold increase over blank media.

**
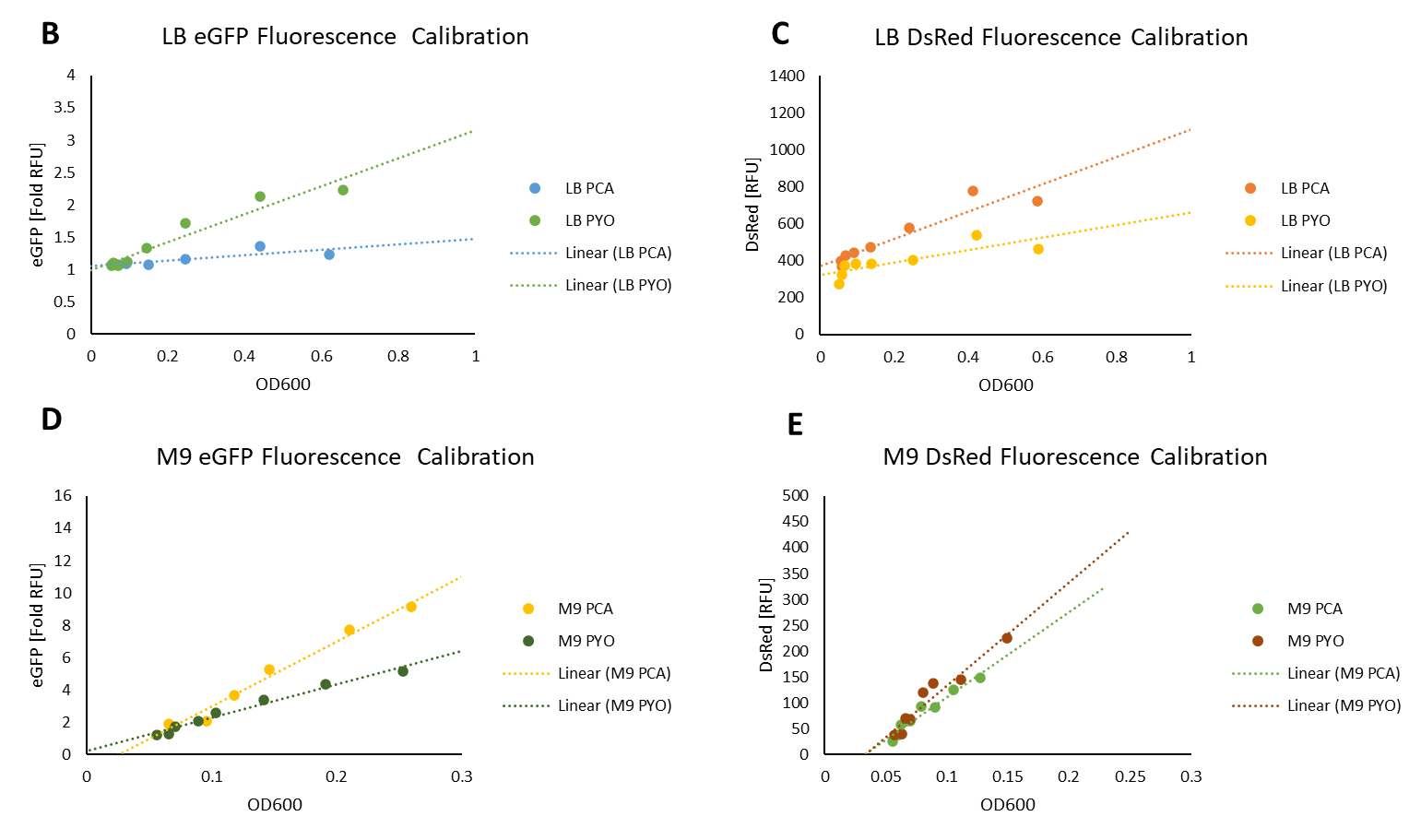
**

**
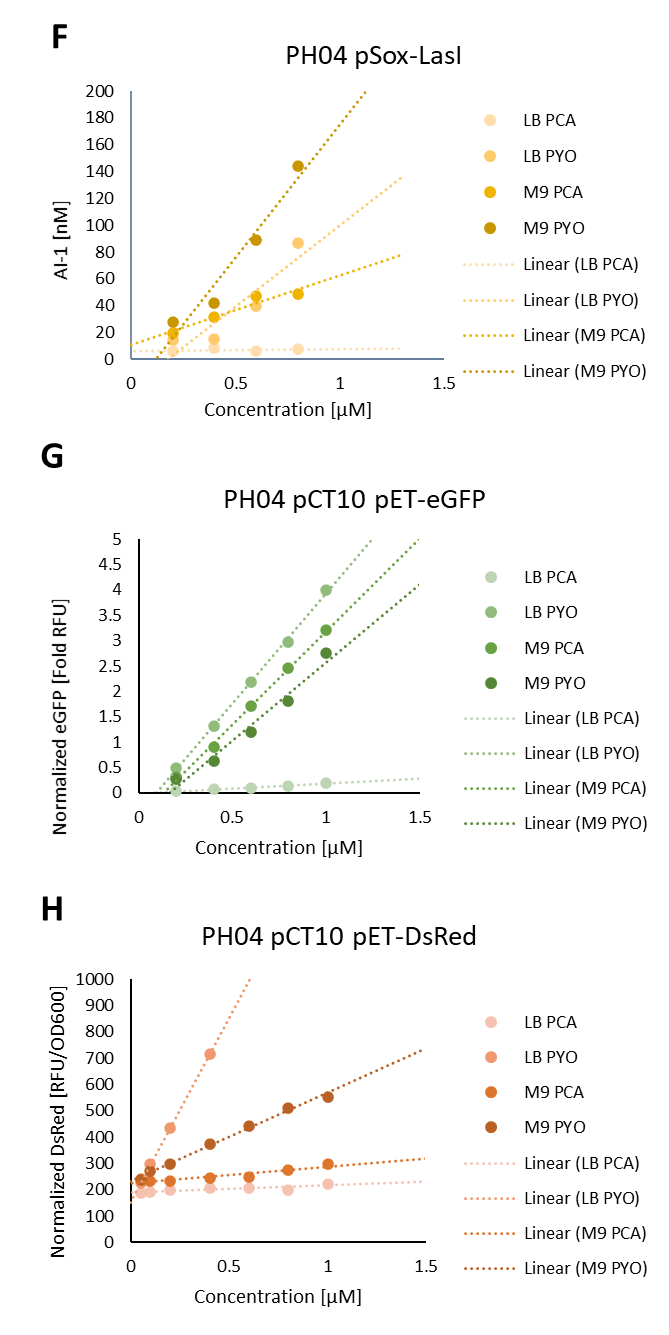
**

**Figure S2: Tri-culture Growth**

Population A (*E. coli* PH04 pZE-phzAG-ptsH-DsRed) and Population B (*E. coli* PH04 pZE-phzMS) were co-cultured together at initial inoculation ratios as indicated on the x-axis and with nanomolar AI-1 concentrations as indicated in the legend. After overnight growth, 20 µL of the PYO producing co-culture (~1.5 OD_600_) was added to a culture of ~0.2 OD_600_ Population C (PH04 pCT10-pET-eGFP) with 200 µL final volume in fresh media. The tri-strain culture was then incubated for 4.5 hours, measuring OD_600_ every 30 minutes.

**
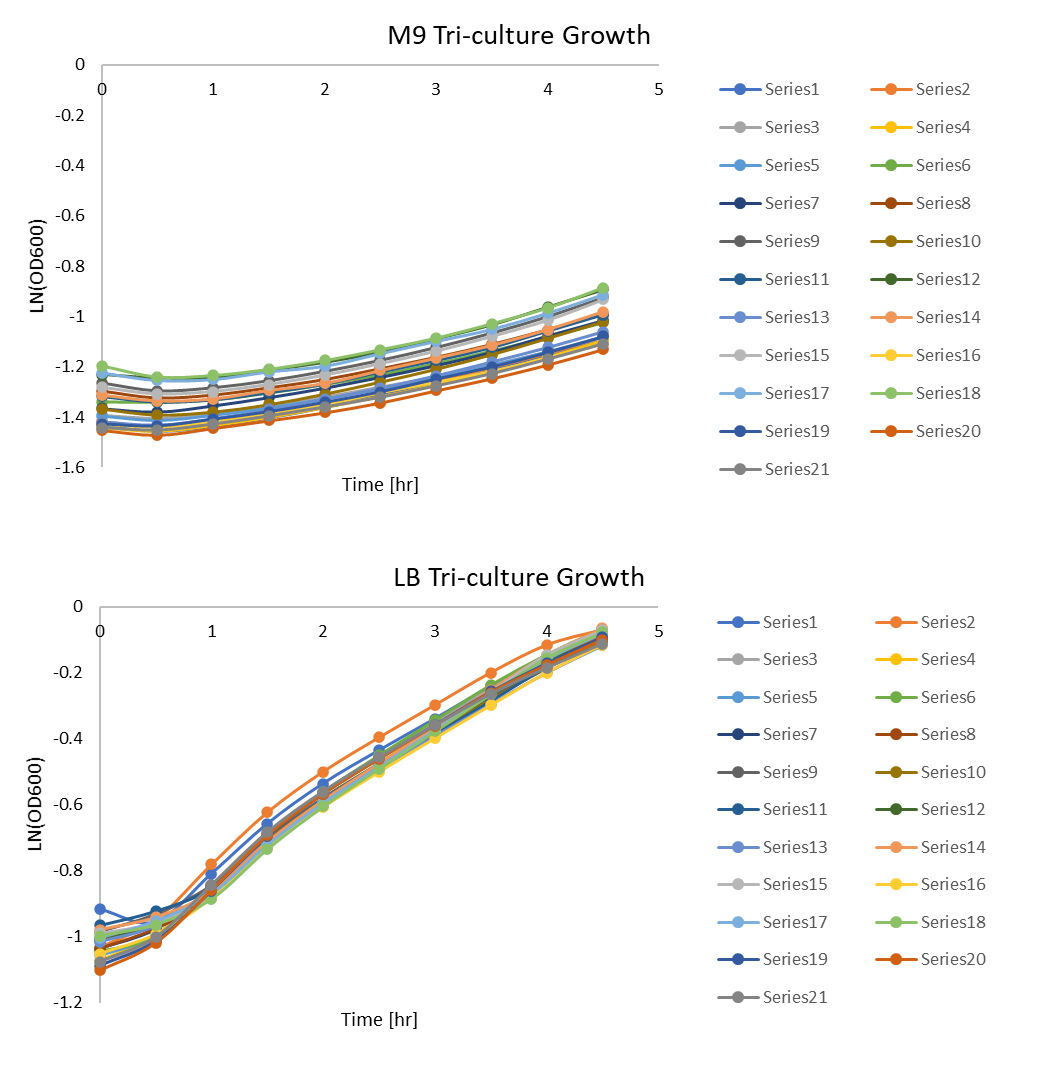
**

**Table S3: Tri-culture Growth Rates**

The average growth rates and their standard error for tri-cultures were calculated from timepoints spanning 1 to 4.5 hours (**Figure 2)**.

**
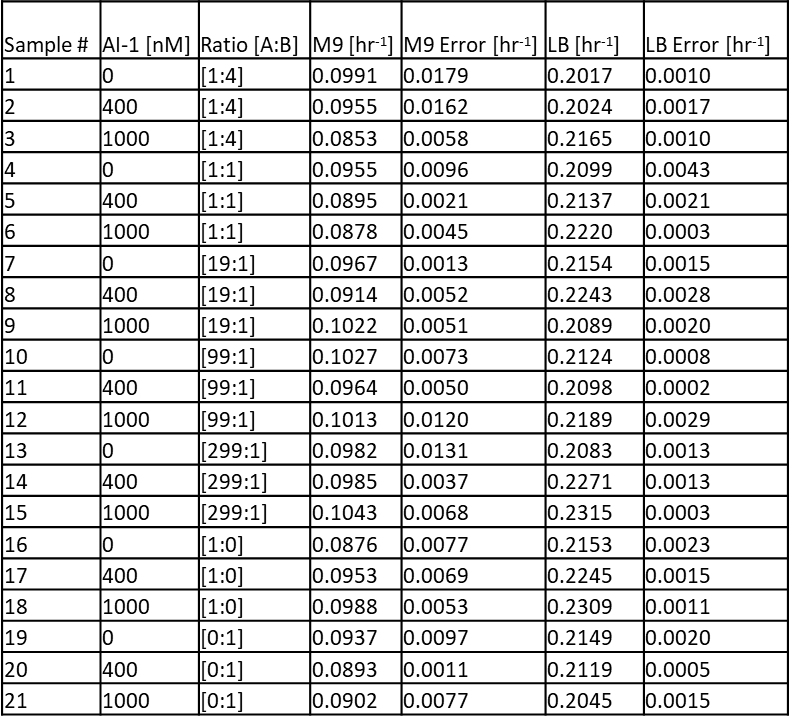
**

**References**

1. Stephens, K., Pozo, M., Tsao, C.Y., Hauk, P. & Bentley, W.E. Bacterial co-culture with cell signaling translator and growth controller modules for autonomously regulated culture composition. *Nature communications* **10**, 4129 (2019).

2. Wang, L., Hashimoto, Y., Tsao, C.Y., Valdes, J.J. & Bentley, W.E. Cyclic AMP (cAMP) and cAMP receptor protein influence both synthesis and uptake of extracellular autoinducer 2 in Escherichia coli. *Journal of bacteriology* **187**, 2066-2076 (2005).

3. Wang, S., Tsao, C.Y., Motabar, D., Li, J., Payne, G.F., Bentley, W.E. A Redox-based Autoinduction Strategy to Facilitate Expression of 5xCys-tagged Proteins for Electrobiofabrication. *Frontiers in Microbiology* **12**, 1473 (2021).

4. Tschirhart, T. et al. Electronic control of gene expression and cell behaviour in Escherichia coli through redox signalling. *Nature communications* **8**, 14030 (2017).
